# Wellness4Students Program to Mitigate Stress, Anxiety, and Depression Among Makerere University Students in Uganda: Protocol for a Longitudinal Study

**DOI:** 10.2196/84637

**Published:** 2026-03-30

**Authors:** Stephen Ojiambo Wandera, Olivia Nankinga, Patricia Ndugga, Christabellah Namugenyi, Claire Ashaba, Noeline Nakasujja, Rosco Kasujja, Belinda Agyapong, Vincent Israel Opoku Agyapong

**Affiliations:** 1 Department of Population Studies, School of Statistics and Planning, College of Business and Management Sciences, Makerere University, Makerere Hill Road, Kampala, 256, Uganda, 256 754976879; 2 Department of Planning and Applied Statistics, School of Statistics and Planning, College of Business and Management Sciences, Makerere University, Kampala, Uganda; 3 Department of Psychiatry, School of Medicine, College of Health Sciences, Makerere University, Kampala, Uganda; 4 Department of Mental Health & Community Psychology, School of Psychology, College of Humanities and Social Sciences, Makerere University, Kampala, Uganda; 5 Department of Psychiatry, Faculty of Medicine and Dentistry, University of Alberta, Edmonton, AB, Canada; 6 Department of Psychiatry, Faculty of Medicine, Dalhousie University, Halifax, NS, Canada

**Keywords:** stress, anxiety, depression, students, Wellness4Students, mental health, Uganda, Africa, email, WhatsApp

## Abstract

**Background:**

Research indicates that university students experience higher levels of stress, anxiety, and depression (SAD) than the general population. In Uganda, existing psychological interventions for addressing SAD among students are primarily delivered face to face, which limits effective diagnosis and treatment due to stigma, a shortage of counselors, and long waiting times, which contribute to significant unmet mental health needs. Consequently, there is an urgent need for innovative approaches to improve access to mental health services.

**Objective:**

This study aims to assess the feasibility and effectiveness of a supportive SMS text messaging program (Wellness4Students [W4S]) in reducing the prevalence of SAD among university students in Uganda.

**Methods:**

This study has a quantitative longitudinal research design. We will target 4494 undergraduate students aged 18 to 24 years who will be recruited from Makerere University, Uganda. Through advertisements on social media platforms (WhatsApp and X [formerly known as Twitter]) and websites, students will receive a link to the W4S website with information about the intervention. Eligible students will voluntarily self-subscribe to the W4S program by providing their email addresses. The subscription will be an indicator of consent required for participation in receiving the intervention. Subscribers will receive daily supportive messages for 6 months. The data will be collected from subscribers at baseline (program initiation), 1 month, 3 months (midpoint), and 6 months (program completion) through web-based surveys. Students will complete questionnaires regarding SAD and well-being. We will conduct both descriptive and inferential statistical analyses using STATA version 17.

**Results:**

The results of this study are expected within 1 year after program initiation. The data will be collected from February 2026 through October 2026. The development of the W4S app and the survey instrument has been completed. The cocreation of mental health messages with 15 undergraduate students and 2 university psychologists has been completed as of March 23, 2026. The roll-out of the program is scheduled for March 24, 2026.

**Conclusions:**

The W4S intervention is feasible. The W4S program will provide essential insight into the prevalence and correlates of SAD among Makerere University students. We will identify students who are most at risk of poor mental health outcomes.

## Introduction

### Background

Mental health is a critical component of overall well-being. Mental health disorders, such as stress, anxiety, and depression (SAD), have been linked to serious health conditions [1], including cardiovascular diseases, cancer, diabetes, respiratory diseases, substance abuse, chronic pain, sleep disorders, and heart diseases [2]. Globally, mental health disorders are among the most burdensome health problems, accounting for more than 970 million cases [3,4]. According to the United Nations Children’s Fund, more than 13% of young people are affected worldwide [5]. Anxiety and depression alone comprise about 40% of these cases [5]. While these statistics reflect a worldwide crisis, the burden is disproportionately higher in low- and middle-income countries, where mental health infrastructure is underresourced and access to care is critically limited [2,6,7].

In sub-Saharan Africa, this disparity is glaring. The continent has fewer than 1.5 mental health workers per 100,000 population, compared to a global median of 9, and governments allocate less than 1% of health budgets to mental health [2,4,8]. This scarcity is linked to the treatment gap, estimated at over 75% for common mental disorders in sub-Saharan Africa, leaving the vast majority without any form of care [9]. This systemic neglect exacerbates vulnerabilities among populations already facing socioeconomic challenges, including university students.

Evidence consistently indicates a higher prevalence of SAD among university students compared to the general population [10-12]. Multiple meta-analyses indicate that university or postsecondary students have far higher pooled rates of depression and anxiety than the general or same-age population. For instance, a large pre-COVID meta-analysis estimated depression prevalence among university students at 30.6%, far above the global adult prevalence of 12.9% [13]. A more recent global review (64 studies; 100,187 students) found a pooled prevalence of depression at 33.6% and anxiety at 39% among college students [14]. An umbrella review of anxiety in college or university students across all world regions reported a median anxiety prevalence of 32% (range 7.4%‐55%) [15]. A systematic review covering 40 countries found a prevalence of 26.1% for depression and 24.5% for anxiety among university students and explicitly contrasted this with much lower World Health Organization estimates for the general population (depression: 4.4%, anxiety: 3.6%), concluding that disorders “affect a much higher percentage of university population than the general one.” The transition into higher education presents a key developmental period marked by academic pressure, social reorientation, and often financial strain, all of which can significantly impact mental health [16]. These problems not only cause personal distress but also negatively affect students’ quality of life and academic performance [17].

Research across East Africa highlights the scale of the issue, though prevalence estimates vary due to methodological heterogeneity in assessment tools and timing. In Tanzania, a study reported a prevalence of depression of 22% among undergraduates [17], while in Kenya, the prevalence reached 41% [18]. In Uganda, a systematic review found that depression among university students ranged from 0.4% to 80% [16], a range underscoring the need for standardized measurements. A study among medical students at Makerere University found a prevalence of 22% [19]. The COVID-19 pandemic remarkably exacerbated this situation; during the lockdowns, one study among Ugandan students reported alarmingly high rates of depression (81%), anxiety (98%), and stress (78%) [20]. This dramatic increase aligns with global findings of a “mental health pandemic” secondary to COVID-19, particularly affecting youth in educational settings [16].

The determinants of SAD among students are multifaceted, arising from an interplay of sociodemographic, academic, and behavioral factors. Key sociodemographic factors include gender, age, and low socioeconomic status [20-22]. Academic and institutional stressors include demanding programs of study, academic pressure, and the transition to university life [23,24]. Behavioral factors include substance use, excessive social media engagement, and relationship problems [20-22]. The “campus culture” and institutional climate, including perceptions of support (or lack thereof) from faculty and administration, have also been identified as significant moderators of student mental health [20-22].

Despite this significant and multifaceted burden, the treatment gap in Uganda remains vast due to systemic, context-specific barriers. The deeply ingrained stigma, often rooted in cultural taboos and spiritual beliefs that attribute mental illness to moral failure or supernatural causes, deters help-seeking [25,26].

Qualitative studies in Uganda specifically cite fear of gossip and being labeled “kizibu” (mad) as primary reasons students avoid formal services. This is mainly linked to low mental health literacy and a crippling shortage of services: Uganda has fewer than 2 psychiatrists, with counselor-to-student ratios often exceeding 1:10,000 [2,4,8,19,27]. Structural and policy gaps limit care, as mental health remains chronically underfunded within national and institutional budgets [25,28]. Yet, these countries experience a significant burden of mental health disorders, with over 24% of individuals qualifying for psychiatric diagnosis [10,16,21,23,27,29-31].

Consequently, current psychological interventions in Uganda are primarily limited to unsustainable person-to-person approaches, hindered by stigma, cultural taboos, long wait times, policy gaps, and insufficient resource allocation [16,19,20,23,27,32]. Available alternative interventions are often Western-based and not tailored to the unique sociocultural and economic context of Ugandan students [33-36]. This lack of cultural adaptation can reduce intervention engagement, relevance, and effectiveness, a known challenge in global mental health transitions [37,38]. There is, therefore, a critical need to develop, adapt, and test scalable, low-cost, and contextually relevant interventions that can bypass traditional barriers to care.

Digital mental health interventions, delivered through widely accessible platforms like email and WhatsApp, offer a promising pathway. Systematic reviews suggest that digital interventions, particularly those based on cognitive behavioral therapy (CBT) principles delivered via text or app, are effective in reducing symptoms of depression and anxiety in young adults [39,40]. They can provide stigma-free and consistent support, directly addressing gaps in accessibility and human resources. Their scalability makes them particularly suitable for low-resource settings. Preliminary studies in other African contexts, such as SMS-based support for perinatal depression in Kenya, demonstrate feasibility and high acceptability [41,42]. This study responds to this urgent need by assessing the feasibility and effectiveness of a supportive SMS text messaging program (Wellness4Students [W4S]) in reducing the prevalence of SAD among university students in Uganda.

### W4S Email and WhatsApp Messaging Intervention

The W4S program is a daily, supportive email and WhatsApp messaging intervention designed to mitigate symptoms of SAD and promote well-being. It is part of the broader ResilienceNHope suite, supported by a web-based platform and implemented through the Global Psychological eHealth Foundation. The initial content is developed by mental health professionals using CBT principles. Crucially, for this study, the program will be contextually adapted. The initial message database contains generic content that will serve only as a starting point for adaptation. Through participatory cocreation workshops with students at Makerere University, this content will be linguistically, culturally, and contextually adapted to ensure that metaphors, proverbs, and messages address specific contextual stressors for the students. This participatory process is central to the intervention’s design, ensuring it is not merely imported but is collaboratively shaped by its intended users [25,28,33,43].

Through a partnership with Makerere University, students will be able to self-enroll online. Upon registration, they will receive daily supportive, noninteractive (one-way) messages for 6 months, with the nature of the service clearly explained at the outset. Each day’s email and WhatsApp message will be unique, drawn from a large message database.

Examples of messages (not yet adapted) to be delivered to subscribers of the W4S email and WhatsApp messaging program include the following:

- What you do today will determine how you are tomorrow. Rise up and take advantage of whatever opportunities today presents, and success will be yours tomorrow.
- There are 2 days in the week we should not worry about, yesterday and tomorrow. That leaves today, live for today.
- There are no surprises in store. You are in charge of the successes that await you. There may be some challenges, but you have the power to overcome them.

### Aims of the Study

This study aims to determine the prevalence and correlates of SAD among university students in Uganda. It also aims to determine if daily supportive text messages can help reduce the prevalence of SAD and improve well-being among university students.

### Specific Objectives

This study has the following objectives: (1) to determine the prevalence and correlates of SAD among university students in Uganda, (2) to investigate the effectiveness of the daily W4S supportive messages in reducing levels of SAD and in improving well-being among university students in Uganda, and (3) to investigate the participants’ experience and satisfaction associated with the W4S daily SMS text messaging support.

## Methods

### Study Design

The study used a quantitative longitudinal study design. It will be implemented in three phases: (1) the cocreation and adaptation of the intervention; (2) the baseline survey; and (3) follow-up surveys conducted at 1 month, mid-term follow-up at 3 months, and an endline at 6 months. The cocreation phase was completed during the recess term (July 2025). The next 2 phases, baseline and follow-ups, will be implemented between February 2026 and October 2026. Recruitment will begin in February 2026 and will last for 3 months up to April 2026. There will be an observation period of 6 months for the enrolled participants [28,33,43].

The email and WhatsApp messaging intervention was cocreated and adapted by the study team together with purposively selected students and identified stakeholders. Thereafter, a baseline survey will be conducted before recruitment to estimate the prevalence of SAD. Students at Makerere University will be invited via email and WhatsApp to self-subscribe to the W4S program. All participants who subscribe to the intervention will receive a link to the online baseline survey, daily supportive messages for 6 months, and the follow-up survey links at 1-, 3-, and 6-month postenrollment time points [33,43].

The rationale for the nonrandomized design is based on resource constraints. We have funding to implement a pilot survey and use the preliminary results to apply for a bigger grant to implement a fully randomized controlled trial.

### Study Setting

The study will be conducted among Makerere University undergraduate students. The university has a population of over 35,000 students [44]. Since the baseline survey and the follow-up will be conducted virtually, general assumptions are made that all students own smartphones, have an email and WhatsApp, and can access free Wi-Fi at Makerere University.

### The Intervention: Cocreation of Email and WhatsApp Messages

For this protocol, the intervention to be delivered to the students at Makerere University is the ResilienceNHope program. The ResilienceNHope suite of interventions delivers daily supportive messages, often accompanied by images and links to mental health literacy resources, to its subscribers. A total of 15 students (8 female and 7 male) were selected across years of study (year 1, year 2, and year 3) at the College of Business and Management Sciences, Makerere University, to ensure representativeness of the cocreation team and to avoid potential biases in the adaptation process.

Supportive text messaging programs based on CBT principles, such as the W4S program within the ResilienceNHope suite, are among the most highly recommended digital interventions for SAD and have been successfully implemented in both Canada and Zambia [33-35,43]. The study team, together with selected undergraduate students and identified stakeholders, will adapt the W4S program, specifically tailoring messages and images to the Ugandan undergraduate university students’ population [43].

There are several adaptation frameworks. These include the ADAPT-ITT (Assessment, Decision, Adaptation, Production, Topical experts, Integration, Training, Testing), intervention mapping (IM) framework, and framework for reporting adaptations and modifications to evidence (FRAME), among others [38,45,46]. For this study, we will adapt the W4S program [33,43] using the IM adaptation framework [47]. The IM adaptation framework has 6 steps. First, establish a detailed understanding of the health problem, the population at risk, the behavioral and environmental causes, and the determinants of these behavioral and environmental conditions and assess available resources. Second, describe the behavioral and environmental outcomes, create objectives for changes in the determinants of behavior and environmental causes, and specify the targets of the intervention program. Third, identify theory- and evidence-based behavior change methods that influence the determinants and translate these to practical applications that fit the intervention context. Fourth, combine the intervention components into a coherent program that uses delivery channels that fit the context. Fifth, develop implementation strategies to facilitate the adoption, implementation, and maintenance of the program. Finally, plan both process and outcome evaluation to assess program implementation, efficacy, or effectiveness [47].

The adaptation is expected to improve the feasibility and acceptability of the W4S intervention [33-35,43]. The cocreation and adaptation will be done when students are in the recess term period (between June and July).

### Data Collection

An online baseline survey will be conducted to assess the mental health of the students.

A link to the baseline survey will be sent to the students through their email and WhatsApp. Clicking on the link to the baseline survey will indicate consent required for participation in the survey. The data collection portal is secure and password-protected with dual authentication, which minimizes data breaches. In addition to the demographic questions, participants will fill out the online screening tools for assessing SAD and well-being. It is on this basis that they will be diagnosed (to have or not to have) with at least one of the 3 outcomes [8,48-50]. The app is programmed to produce summaries of the total number of subscribers at any given point in time and how many have completed the different phases.

All participants will be able to view their scores for each mental health outcome (SAD). Depending on the severity of the mental health scores, recommendations will be provided to participants. All students, whether diagnosed with SAD or not, will have the option to enroll in the W4S program and will be made available through a postbaseline subscription link. The baseline survey and intervention enrollment will be conducted from March 2026 to cover the second semester of the academic year 2025/2026 at Makerere University.

### Baseline Survey

A structured baseline survey questionnaire will be developed to measure students’ mental health outcomes. The questionnaire will include the 10-item Perceived Stress Scale (PSS-10), 7-item Generalized Anxiety Disorder (GAD-7) scale, 9-item Patient Health Questionnaire-9 (PHQ-9) scale, and WHO-5 (World Health Organization-Five Well-Being Index) scale to measure SAD and well-being [20,49,51-53]. These tools have been validated for making comparisons between population subgroups [35]. The questionnaire will also include sociodemographic and economic variables, including age, relationship status, religion, orphanhood status [54], college, sponsorship status, working status, living arrangement, year of study, and current academic performance among others. Participant data will be collected online through self-administered questionnaires on the ResilienceNHope platform [43]. The data will be hosted online on the ResilienceNHope server and downloaded for analysis.

### Follow-Up Surveys

A follow-up survey questionnaire will be used to assess improvements in the students’ mental well-being using validated scales for SAD, using the PSS-10, GAD-7, and PHQ-9. These surveys will be conducted at 1 month, 3 months (mid-term evaluation), and 6 months of the study (endline evaluation). The app has automated email reminders at 6 weeks, 3 months, and 6 months to boost completion rates. In addition, receiving daily messages with images is an incentive to students, and this was tested during the cocreation process with students.

The questionnaire will also include variables on participants’ satisfaction with the W4S program intervention and suggested improvements and recommendations. Participant data will be collected and hosted online through self-administered questionnaires on the ResilienceNHope web platform [43] and downloaded for analysis. We recognize that retention is and has been a challenge for online surveys; part of the mitigation strategy is to make the survey shorter. The number of questions was reduced to the bare minimum.

### Participants’ Recruitment and Subscription

This study will be conducted among Makerere University students in Kampala, Uganda. We will consider currently enrolled students from year 1 to year 3 of study. We have made an assumption that all university students own smartphones based on a previous study [55]. Internet access is always guaranteed to all university students during the semester when the study will be implemented. We will broadcast information about the study through social media platforms used by university students, including WhatsApp, emails, and Twitter (subsequently rebranded X). We will also share information about the study through posters and by word-of-mouth within accessible groups. We will also engage the staff of the Counselling and Guidance Center at the University Hospital to inform students about this study.

During the broadcast, students will be informed about the possibility of being enrolled in a digital intervention program (W4S program). The study will be launched through an announcement by the office of the Dean of Students of Makerere University, the Students’ Guild, and the Director of Makerere University Hospital. All interested students will be provided with a link to the W4S website to self-subscribe to the study. Potential respondents will be asked to register for the study with their active emails and also consent to being part of the study. Once they have electronically consented to the study, they will receive another link to the baseline study. The cocreated and adapted messages will be sent to subscribers’ email and WhatsApp daily at 7 AM East African Time. Each subscriber will receive the W4S program daily for 6 months.

### Midterm and Endline Evaluation

The program will be evaluated at 1 month, 3 months (mid-term), and 6 months (endline evaluation) after the students start receiving daily supportive email and WhatsApp messages. The midterm and endline surveys will assess improvements in the students’ mental well-being using validated scales for the primary outcomes: SAD [20,48-51,53].

### Outcomes and Measures

Primary outcome measures at baseline, 1 month, 3 months, and 6 months will be the mean difference in scores on the PSS-10, GAD-7 scale, and PHQ-9 [49,56-58]. Prevalence estimates for SAD will be calculated as the total number of undergraduate students assessed as likely to have a particular condition divided by the total number of students who completed the baseline survey. In addition, proportions for survey uptake, response rates, and completion rates will be calculated. Finally, the outcomes related to the feasibility or acceptance of the tool, along with measures of engagement and adherence, will be collected at the end of the follow-up survey.

### Sample Size Estimation

The total student population at Makerere University, Kampala, Uganda, is about 35,000 [41]. It is estimated that the sample size needed for our prevalence estimates with an expected SAD prevalence of 50% [10,12], a 95% interval, and a 2% margin of error for moderate-to-high stress, moderate-to-high anxiety, and moderate-to-high depression among Makerere University students will be 2247. A sample size calculator was used to determine the sample size [28,59,60].

Based on the previous maximum survey completion rates of 50% observed in similar programs, a minimum of 4494 students will be enrolled in the W4S email and WhatsApp messaging program over 6 months to obtain an estimated 2247 students’ completion at baseline. To achieve a power of 80% (*β*=.2) and a 2-sided significance level of 5% (*α*<.05) and to detect an effect size of 30% before and after the intervention, a sample size of 90 would be needed [49].

### Statistical Analysis

Once data are downloaded from the W4S server, the data from the baseline and follow-up surveys will be exported to STATA version 17 for data analysis. Observations with more than 50% missing responses will be omitted from the dataset. Otherwise, missing data on individual variables will be handled using multiple imputation with baseline and postbaseline variables [61].

At baseline, frequency distributions will be used to summarize categorical variables, while means and SDs will be used for continuous variables. Chi-square tests and/or *F* statistics will be used to measure the association between demographic characteristics and SAD variables. Multivariable logistic regressions will be used to determine the correlates of SAD among MAK undergraduate students. We plan to conduct rural-urban disaggregated analysis to account for diversity among the students.

Paired sample 2-tailed *t* tests and proportion tests will be used to assess significant differences in mean scores and prevalence rates of SAD at baseline, 1 month, 3 months, and 6 months. Profile plots of mean scores will be generated while controlling for background characteristics. All findings will be reported at 95% CI with a significance level of 5% (*P*<.05). The data will be analyzed using Stata version 17.

### Data Quality Assurance

At each stage of data collection, we will implement checks to ensure that we collect high-quality data, enabling us to generate accurate insights. A 3-member data quality committee will be established, which will be responsible for checking quantitative data that are uploaded to the server. All data will be checked for consistency and accuracy. Any errors and inconsistencies will be immediately scrutinized for corrective action. Also, we will use algorithmic checks, such as Mahalanobis distance for multivariate outliers, integrated into STATA scripts to ensure data integrity and reproducibility [62].

### Ethical Considerations

Ethical approval for this study was granted by the AIDS Support Organisation Research Ethics Committee (TASO-2025‐2141) and the Uganda National Council of Science and Technology (UNCST SS4200ES).

Participation in the study will be voluntary, where students will self-subscribe to receive daily supportive emails and WhatsApp messages for 6 months. All respondents will be assured of confidentiality, and participants will be made aware that informed consent will be implied when they complete and submit the survey. Privacy and confidentiality will be guaranteed. The data will be anonymized and deidentified, and protective measures will be taken to safeguard participant information. All students will be compensated with a one-off airtime compensation of CAD $5 (equivalent to US $3) after completing the surveys.

Potential participants will be informed of their right to withdraw from the study. The online questionnaire will automatically end upon their declining to participate in the study. The dataset from the website is programmed to be downloaded without identification particulars (deidentified), and therefore, no personal information will be publicized. The particulars of the respondents are only available to the principal investigator and the core research team.

Students with adverse mental health outcomes will be referred to the university’s Counselling and Guidance Center or advised to seek professional help.

### Hypotheses

We hypothesize that the W4S email and WhatsApp messaging program will reduce the prevalence and severity of SAD among Makerere University students. We also hypothesize that the prevalence of moderate-to-high stress, moderate-to-high anxiety, and moderate-to-high depression would be comparable to the prevalence of these conditions reported among university students in East Africa (Kenya and Tanzania) and in other jurisdictions. Finally, we hypothesize that demographic factors, socioeconomic factors, organizational factors, and class size will be associated with SAD among students.

## Results

The results will be reported following the SPIRIT (Standard Protocol Items: Recommendations for Interventional Trials) checklist [63]. The W4S email and WhatsApp messaging program was anticipated to be launched in September 2025. There were some delays, and the enrollment will start by March 24, 2026. Enrollment is expected to continue for 6 months up to September 2026. Data collection will continue for another 6 months. The results of the study will be disseminated to stakeholders in the education sector in Uganda, East African countries, and globally through workshops, conference presentations, and peer-reviewed publications.

The progress to date is that ethical approval has been secured. We conducted a cocreation of intervention messages. A pilot survey was conducted with about 20 students. We are going to implement the enrollment and the intervention during the second semester of the academic calendar (February-May 2026). We will implement a survey from March 2026 at different time intervals.

## Discussion

### Principal Findings

SAD may indirectly hinder university students’ academic performance, overall lifestyle, psychological safety, and well-being, as well as lead to other physical health problems. Given their demanding schedules, students need innovative, anonymized, accessible, convenient, and cost-effective programs to support their mental health. This protocol proposes the use of supportive email and WhatsApp messages as a psychological intervention for students experiencing SAD. Those with any of the target outcomes, prior traumatic experiences, or adverse childhood events are expected to benefit particularly from this program. We anticipate a reduction in mental health outcomes, such as reduced SAD among university students at Makerere University.

The study will generate important information on the prevalence and correlates of SAD among students at Makerere University, Kampala, Uganda. It will also provide valuable insights into the application of eHealth approaches for young populations within the education sector. The effectiveness of daily supportive email and WhatsApp messaging in addressing these mental health concerns will be evaluated, offering critical evidence to guide school policies and decisions on psychological interventions for students in low- and middle-income countries.

### Strengths and Limitations

The strengths of this protocol include the following: first, the process of cocreation is a core strength of the intervention. This is a novel approach in low- and middle-income country eHealth interventions. This differentiates our protocol from top-down interventions and highlights participatory science.

Second, we expect that the survey will provide insightful data on the prevalence of SAD among university students. This will support the Counselling and Guidance Center of the university in tailoring interventions to the student community.

However, some limitations are anticipated. First, the outcome measures will be assessed at 1, 3, and 6 months. However, the effects of the intervention beyond 6 months remain unknown. It is also uncertain whether the benefits will decline once the daily email and WhatsApp messages end. Second, although the self-reported scales used to measure mental health variables are standardized, they are not diagnostic tools. Third, the demographics of study participants may not represent those of the wider student body at other universities (public and private or rural and urban), limiting the generalizability of the findings. Additionally, web-based surveys distributed via email and WhatsApp typically have response rates below 20%, which may hinder achieving the target sample size. Nonetheless, this will be the first study in Uganda to examine the prevalence and associated factors of SAD among students using an email and WhatsApp messaging program. We plan to use these preliminary data as a pilot for larger trials and invite collaborative follow-ups.

### Conclusions

This study will provide vital information on the prevalence and correlates of SAD among students at Makerere University. The study will also provide preliminary evidence of the “feasibility of effectiveness” of the W4S (email and WhatsApp messaging) program, W4S, in reducing mental health disorder symptoms. The knowledge gained from this study will significantly impact the promotion of the management of SAD.

The study outcome will help inform policy decision-making for health care resource allocation in support of advocacy for tech equity in the educational sector, directed toward Ugandan students. In addition, it will address the eHealth-policy nexus. The feasibility data could reshape Uganda’s national mental health policy.

## Abbreviations

ADAPT-ITT: Assessment, Decision, Adaptation, Production, Topical experts, Integration, Training, Testing
CBT: cognitive behavioral therapy
FRAME: framework for reporting adaptations and modifications to evidence
GAD-7: 7-item Generalized Anxiety Disorder
IM: intervention mapping
PHQ-9: 9-item Patient Health Questionnaire
PSS: Perceived Stress Scale
SAD: stress, anxiety, and depression
SPIRIT: Standard Protocol Items: Recommendations for Interventional Trials
W4S: Wellness4Students
WHO-5: World Health Organization-Five Well-Being Index
